# AMPK: a novel target for treating hepatic fibrosis

**DOI:** 10.18632/oncotarget.19376

**Published:** 2017-07-19

**Authors:** Zhenxing Liang, Tian Li, Shuai Jiang, Jing Xu, Wencheng Di, Zhi Yang, Wei Hu, Yang Yang

**Affiliations:** ^1^ Department of Cardiothoracic Surgery, The First Affiliated Hospital of Zhengzhou University, Zhengzhou 450052, China; ^2^ Key Laboratory of Resource Biology and Biotechnology in Western China, Ministry of Education, Faculty of Life Sciences, Northwest University, Xi’an 710069, China; ^3^ Department of Biomedical Engineering, The Fourth Military Medical University, Xi’an 710032, China; ^4^ Department of Aerospace Medicine, The Fourth Military Medical University, Xi’an 710032, China; ^5^ Department of Cardiology, Affiliated Drum Tower Hospital of Nanjing University Medical School, Nanjing 210008, China

**Keywords:** 5'-AMP-activated protein kinase, hepatic fibrosis, hepatic stellate cells, fatty liver diseases, adiponectin

## Abstract

Fibrosis is a common process of excessive extracellular matrix (ECM) accumulation following inflammatory injury. Fibrosis is involved in the pathogenesis of almost all liver diseases for which there is no effective treatment. 5'-AMP-activated protein kinase (AMPK) is a cellular energy sensor that can ameliorate the process of hepatic fibrogenesis. Given the existing evidence, we first introduce the basic background of AMPK and hepatic fibrosis and the actions of AMPK in hepatic fibrosis. Second, we discuss the three phases of hepatic fibrosis and potential drugs that target AMPK. Third, we analyze possible anti-fibrosis mechanisms and other benefits of AMPK on the liver. Finally, we summarize and briefly explain the current objections to targeting AMPK. This review may aid clinical and basic research on AMPK, which may be a novel drug candidate for hepatic fibrosis.

## INTRODUCTION

Fibrosis, the formation of excess fibrous connective tissue in organs, is a major cause of morbidity and mortality worldwide. Fibrosis is a reactive or reparative process characterized by excessive synthesis of ECM and decreased parenchymal cells during inflammatory injury [1]. Fibrosis is a ubiquitous pathophysiological process in various organs, including the liver [2, 3], heart [4, 5], lung [6, 7], pancreas [8, 9], kidney [10, 11], bone marrow [12, 13], and skin [14, 15]. Fibrosis caused by mild or transient injury may repair damaged tissues and recover the original structure and function. However, severe or chronic stimuli exceed the regenerative capacity and contribute to ECM hyperplasia. Hans Popper, the founder of the American Association for the Study of Liver Diseases (AASLD), proposed that inhibiting fibrosis may cure most chronic liver diseases (CLD), thereby indicating a direction of treating CLD for future researchers [16, 17]. Hepatic fibrosis, the excessive accumulation of collagen and polyose in the extracellular space (ECS), usually results from hepatitis viruses, biliary obstruction, and fatty liver diseases (FLD). According to the perspective of the American hepatologist Friedman, hepatic fibrosis is the only route of progression from CLD to hepatic cirrhosis [18]. During the advanced stage of hepatic fibrosis, excessive ECM forms scar tissues and contributes to hepatic decompensation, namely, cirrhosis. In modern medicine, there is still no powerful treatment for fibrosis, the occurrence of which in all organs accounts for almost 45% of deaths in the United States. Therefore, numerous studies have been devoted to searching for a novel drug to redeem the deficiency of the existing strategies.

AMPK is a member of serine/threonine (Ser/Thr) kinase family, which is distributed in various organs, such as the liver, heart, brain, lung, kidney, and skeletal muscle [19]. AMPK was extracted and sequenced by Carling et al. in 1994 and identified as a sensor of energy deprivation [20]. AMPK is a heterotrimeric complex of a 63 kDa α subunit, a 38 kDa β subunit, and a 38 kDa γ subunit [21]. AMPK was initially isolated from liver whereas all three subunits are expressed in a wide variety of tissues, including heart, lung, brain, and kidney [22]. Thereinto, the liver mainly expresses the α1, α2, γ1, and γ2 subunits [21]. Studies have suggested that AMPK protects the liver [23], heart [24], lung [25], and kidney [26] against fibrosis. Our previous work has revealed that AMPK is a protective molecule in ischemia [27], vasorelaxation [28], angiogenesis [29], and fluid shear stress [30]. Numerous studies have also identified the significance of AMPK in fibrosis whereas this relationship has not been well discussed. Therefore, we fully reviewed the literature and presented our efforts to interpret the relationship between AMPK and hepatic fibrosis.

Herein, we first introduce the basic background of AMPK and fibrosis and the role of AMPK in hepatic fibrogenesis. Thereafter, we discuss the three phases of hepatic fibrosis, including primary inflammatory injury, activation of hepatic stellate cells (HSC), and ECM secretion, all of which can be ameliorated by AMPK. Third, we summarize potential drugs for targeting AMPK signaling, the possible mechanisms of anti-fibrosis, and opposing actions of AMPK. Altogether, this review highlights recent research and provides an overview of AMPK signaling, which may be helpful in drug design and clinical therapy of hepatic fibrosis.

## AMPK AND HEPATIC FIBROGENESIS

AMPK is a significant molecule that maintains the homeostasis of energy metabolism. The α subunit is composed of a kinase domain, an auto-inhibitor domain (AID), and an α-subunit carboxy-terminal domain (α-CTD) from the N-terminus to the C-terminus, respectively. Phosphorylation of Thr172 at the α subunit increases the activity of AMPK by 2–3 orders of magnitude [31, 32]. AMP or ADP binding promotes the phosphorylation of AMPK and enhances its activity. The AID domain at the α subunit lowers the activity of AMPK in the absence of AMP. Scott et al. discovered that AMP and A769662 can cause synergistic allosteric activation of AMPK even when using kinase that is not phosphorylated on Thr172 [33]. Phosphorylation of the ST loop, a serine/threonine-rich insert of 50–60 amino acids at the α subunit, may downregulate the activity of AMPK [34]. In addition, overexpression of AMPK using an adenoviral constitutively active form of AMPK (adenoviral constitutively active AMPK) significantly promotes the expression of SHP mRNA in primary hepatocytes and ameliorates hepatic insulin resistance [35]

Hepatic fibrogenesis is a complex pathophysiologic process in response to harmful stimuli, especially inflammatory stimuli [36]. Hepatic fibrogenesis consists of three phases, namely, inflammatory injury, regulation of HSC, and ECM secretion. First, inflammatory injury caused by external or internal stimuli activates hepatocytes and induces inflammatory cytokines secretion. Second, secreted cytokines activate HSC and promote proliferation and migration of HSC. Activated HSC can also secrete inflammatory cytokines and convert to myofibroblasts. Third, activated HSC and myofibroblasts secrete a great quantity of ECM that disturbs the normal substance exchange between hepatocytes and hepatic sinusoid, thereby contributing to hepatocyte death, hepatic insufficiency, and even cirrhosis [37].

**Figure 1:**
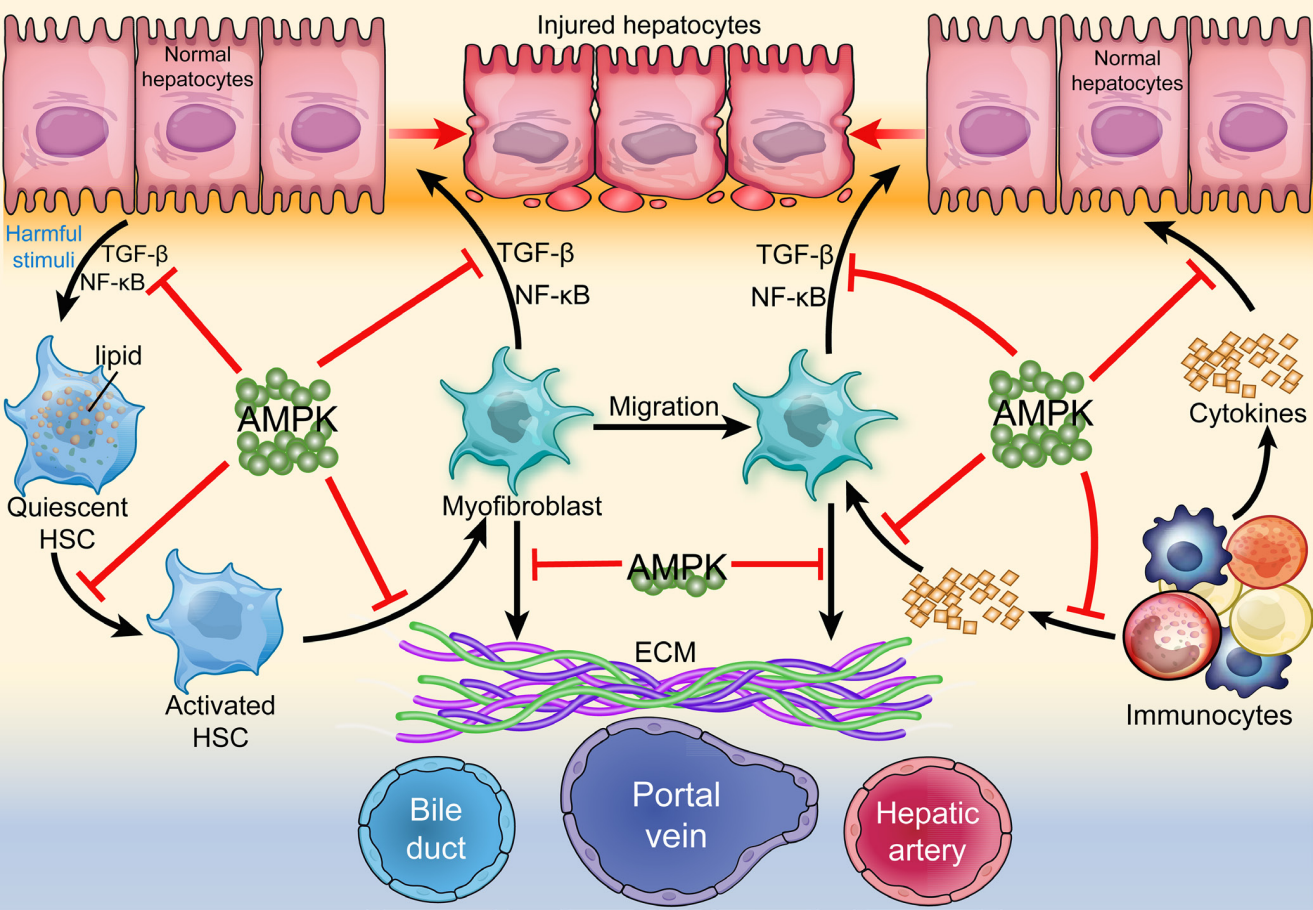
Regulation of AMPK in hepatic fibrogenesis AMPK acts as a central protective molecule against hepatic fibrogenesis. Under harmful stimuli, normal hepatocytes secret TGF-β and NF-κB and induce the transformation of quiescent HSC into activated HSC and myofibroblasts. Immunocytes secret cytokines and activate myofibroblasts, which also secret TGF-β and NF-κB and cause damage to normal hepatocytes. Activated myofibroblasts secret ECM and disturb the normal exchange of material between hepatocytes and portal area, thereby resulting in hepatic fibrosis, inadequacy, and even cirrhosis. All of these process can be ameliorated or delayed by AMPK.

During the process of fibrosis, HSC over-express α-smooth muscle actin (α-SMA) and lose a great quantity of vitamin A, the process of which is similar to fetal hepatic metabolism, indicating fibrosis may induce the re-expression of fetal genes [38]. Experimental or clinical studies have identified a close relationship between AMPK and hepatic fibrogenesis. The activity of AMPK was found to be low in 28 patients with advanced fibrosis/cirrhosis compared to healthy people [39]. Inactivation of AMPK caused by endocannabinoid was reported to inhibit the expression of glucose transporter 2 (GLUT2) on plasmalemma and suppress cellular glucose uptake, which led to impaired glucose metabolism and the promotion of hepatitis C virus (HCV) replication in hepatocytes, thereby increasing the degree of fibrosis [40]. In the mouse carbon tetrachloride (CCl_4_) model, treatment with the AMPK agonist 5-Aminoimidazole-4-carboxamide1-β-D-ribofuranoside (AICAR) suppressed HSC proliferation and collagen-α1 expression and correlated with attenuated hepatic fibrosis and improved liver function [41]. These studies indicate that inactivation of AMPK promotes hepatic fibrogenesis while its activation may restrain this process. (Figure 1).

## ROLES OF AMPK IN THE DIFFERENT PHASES OF HEPATIC FIBROSIS

### Inflammatory injury

During the first phase of hepatic fibrosis, impaired hepatocytes trigger a fibrogenic response to harmful stimuli and recruit inflammatory cells, such as Kupffer cells, nature killer cells (NKCs), lymphoma cells, dendritic cells, and HSC [42, 43]. The common inflammatory inductive factors include hepatitis virus, alcohol, excessive fatty acid, biliary obstruction, and CCl_4_. American hepatologist Scott L. Friedman proposed that hepatic fibrosis is accompanied by inflammation, which is supported by the observation that fibrosis and inflammation always coexist in hepatic pathological sections.

TGF-β is a key regulator of hepatic inflammatory injury, which can be inhibited by AMPK. Lim et al. treated human (LX-2) and rat (CFSC-2G) HSC lines with TGF-β and discovered that AMPK attenuates TGF-β-induced Smad3 interaction with transcriptional coactivator p300 and ameliorates fibrogenic activation. These data demonstrate that AMPK could be a novel target for attenuating inflammatory injury of liver fibrosis [44]. In the mouse CCl_4_ model, it has been shown that ADP355, a short peptide activator of the adiponectin receptor, enhances the expression of AMPK and decreases the levels of TGF-β, which are correlated with inhibition of HSC and fibrosis [45]. Moreover, AMPK attenuates TGF-β-signaling in HCV-infected hepatocytes and inhibits the expression of fibrotic genes, further confirming that AMPK is protective against TGF-β-induced inflammatory injury during hepatic fibrogenesis [46].

AMPK also resists NF-κB-related inflammatory injury during hepatic fibrogenesis. Zhang et al. discovered that ursolic acid enhances the expression of AMPK and restrains the activation of NF-κB in the bile duct ligation (BDL) mouse model, whereas knockout of AMPKα2 reverses the protective effects and exacerbates the degree of fibrosis, revealing that AMPK is a potent drug candidate for BDL-induced hepatic fibrosis by inhibiting NF-κB [23]. This effect was also confirmed by Pan et al, who used rutin, a citrus flavonoid found in a wide variety of plants, to activate AMPK and inhibit NF-κB [47]. In HCV-infected Huh-7.5 cells, Jung et al. reported that AICAR suppresses both NF-κB and TGF-β by activating AMPK. Notably, they also showed that inhibition of NF-κB is attributed to inhibition of TGF-β; TGF-β is an upstream inducer of NF-κB during the inflammatory injury of hepatic fibrosis [46]. Altogether, these findings reveal that AMPK protects against inflammatory injury and delays or attenuates hepatic fibrosis.

### Regulation of HSC

The inhibition of HSC, the main matrix-producing cells involved in the inflammatory and wound-healing response, represents an attractive strategy for the treatment of liver fibrosis. Studies have suggested that AMPK restrains the proliferation, transformation to myofibroblasts, and migration of HSC while promoting apoptosis [48] during hepatic fibrosis.

AMPK is a molecule with powerful anti-proliferative effects against HSC [49]. Liver fibrosis was induced *in vivo* in AMPKα1^–/–^ mice by repeated injection of CCl_4_. In AMPKα1^+/+^ model, stimulation of AMPK activity by AICAR inhibits HSC proliferation and collagen αI expression whereas the degree of fibrosis is obvious in AMPK α1^-/-^ mice, suggesting that defective AMPK activity would enhance hepatic fibrogenesis and fibrosis [41].

AICAR promotes the expression of AMPK and inhibits the proliferation of myofibroblasts derived from HSC in the mouse CCl_4_ model, which is accompanied by reduced levels of collagen I and fibrosis [41]. Li and colleagues used the same model and reported that berberine inhibits the proliferation of HSC and the expression of α-smooth muscle actin (α-SMA). Notably, they discovered that the anti-proliferative effects of AMPK are dose- and time-dependent [50]. Adiponectin also induces the expression of AMPK and restrains the proliferation of HSC in the mouse CCl_4_ model, thereby ameliorating the degree of fibrosis [39].

Transformation of HSC to myofibroblasts is a pro-fibrogenic process that increases α-SMA and decreases stored lipid. α-SMA is a classic feature of activated HSC and myofibroblast. AICAR reduces the levels of α-SMA in the mouse CCl_4_ model by activating AMPK [41]. Thymoquinone, a phytochemical compound for treating liver diseases, significantly activates AMPK and restrains the expression of α-SMA, which is concomitant with reduced levels of collagen and fibrosis [51]. The reduced production of lipids is also induced by HSC transformation. Curcumin-induced AMPK activation promotes the expression of genes that are relevant to lipid accumulation and increases intracellular lipids in HSC, thereby restraining the transformation of HSC to myofibroblasts [52].

Migration is another feature of activated HSC. Macrophage migration inhibitory factor (MIF) is a pleiotropic inflammatory cytokine involved in the migration of HSC. Heinrichs and colleagues reported that MIF promotes the phosphorylation of AMPK and restrains platelet-derived growth factor (PDGF)-induced HSC migration. Interestingly, they discovered that these protective effects are diminished in CD74 knockout mouse, suggesting that the anti-migratory function of MIF is mediated by the CD74/AMPK signaling pathway [53]. Moreover, AMPK-induced anti-migratory actions have also been reported in other studies [39, 54–56].

Additionally, AMPK induces HSC apoptosis. Wang et al. discovered that berberine enhances the expression of AMPK and induces HSC apoptosis through reducing Bcl-2/Bax ratio and subsequently the caspase pathway. Interestingly, they discovered that these effects are mediated by mitochondrial membrane potential loss [57]. Adiponectin-induced AMPK activation promotes apoptosis and inhibits proliferation of HSC by promoting iNOS/NO pathway [55]. Taken together these data show that AMPK can positively reverse the pathological alterations of HSC to inhibit hepatic fibrosis.

### ECM secretion

The last phase of hepatic fibrogenesis is ECM secretion, including collagen and proteoglycan. In liver, ECM secretion is a dynamic process, which is accurately controlled by intracellular genes. The main functions of the ECM are mechanical support, connection, and cell signal transduction [58]. Severe or chronic inflammation induces excessive secretion of ECM, which may form gitterfasern and exacerbate hepatocyte injury. Adiponectin activates AMPK and restrains the expression of collagen I in HSC, concomitant with decreased levels of fibrosis. Notably, AMPK-inhibited collagen remodeling was also discovered in this study, further confirming that AMPK suppresses ECM secretion and hepatic fibrogenesis [55]. Thymoquinone restrains ECM secretion in the thioacetamide-induced mouse hepatic fibrosis model via LKB1/AMPK signaling and attenuates the levels of fibrosis [51]. Other studies also demonstrated that AMPK reduces ECM secretion during hepatic fibrogenesis [41, 45, 59–62] where a detailed elucidation of the mechanisms was beyond the scope of the studies. Together, these findings reveal that AMPK attenuates hepatic fibrosis by inhibiting primary inflammatory injury, ECM secretion, and the induction of HSC. (Table 1).

**Table 1:** Roles of AMPK on different phases of hepatic fibrosis

Phases	Models	Effects	Year	Reference
Inflammatory injury	Human (LX-2) and rat (CFSC-2G) HSC	AMPK attenuates TGF-β-induced Smad3 interaction with transcriptional coactivator p300 and ameliorates fibrogenic activation.	2012	[44]
Inflammatory injury	Mouse CCl_4_ model	ADP355 enhances the expression of AMPK and decreases the levels of TGF-β, correlated with inhibition of HSC and fibrosis	2014	[45]
Inflammatory injury	BDL mouse model	Ursolic acid and rutin enhances the expression of AMPK and restrains activation of NF-κB	2015, 2014	[23, 47]
Inflammatory injury	HCV-infected Huh-7.5 cells	AICAR suppresses both NF-κB and TGF-β by activating AMPK.	2015	[46]
Inhibition of HSC	Mouse CCl_4_ model	AMPK inhibits the proliferation of HSC and the expression of α-SMA	2014, 2010	[41, 50]
Inhibition of HSC	Mouse thioacetamide model	Thymoquinone significantly activates AMPK and restrains the transformation of HSC to myofibroblasts	2014	[51]
Inhibition of HSC	CD74 knockout mouse	MIF-induced HSC migration was detected when CD74/AMPK pathway was blocked	2011	[53]
Inhibition of HSC	BDL mouse model	Berberine enhances the expression of AMPK and induces HSC apoptosis through reducing Bcl-2/Bax ratio and subsequent caspase pathway	2016	[57]
ECM secretion	Sprague Dawley rat HSC	Adiponectin activates AMPK and restrains the expression of collagen I in HSC, concomitant with decreased fibrotic levels.	2015	[55]

## PROTECTION OF AMPK AGAINST HEPATIC FIBROSIS BY VARIOUS CAUSES

### HCV

HCV is a small single-stranded RNA virus that causes chronic hepatitis and cirrhosis [63]. Treatment with endocannabinoids decreases the levels of AMPK and promotes HCV replication in hepatocytes, thereby exacerbating the levels of fibrosis and the metabolic disorder of the hepatocytes. However, treatment with a cannabinoid receptor antagonist activates AMPK, improves disorders of glucose metabolism, inhibits viral genome replication, and reverses hepatic fibrosis [40]. Small heterodimer partner (SHP), an atypical orphan nuclear receptor, can activate AMPK and suppress HCV replication by inhibiting TGF-β and NF-κB [46]. Jung and colleagues, using AICAR, discovered that it inhibits HCV replication and fibrosis via AMPK-induced anti-inflammatory pathway [46]. However, current reports on AMPK and HCV-induced fibrosis are insufficient, and this area requires further studies to elucidate this problem.

### FLD

FLD, which is manifested as accumulated vacuoles of triglyceride in hepatocytes, include non-alcoholic fatty liver diseases (NAFLD) and alcoholic fatty liver diseases (AFLD) [64, 65]. FLD are dependent risk factors of hepatic fibrosis and cirrhosis. Studies have suggested that chronic exposure to alcohol may inhibit the activity of AMPK, enhance the activity of acetyl-CoA carboxylase (ACC), and decrease the levels of malonyl coenzyme A, thereby contributing to FLD and fibrosis [66–68].

Nonalcoholic fatty liver disease (NAFLD) includes simple steatosis, nonalcoholic steatohepatitis (NASH), hepatic fibrosis, and cirrhosis. There is approximately 30% of the US population with NAFLD annually [69]. AMPK also attenuates lipid accumulation in murine hepatocytes and mice fed a high fructose diet, which correlates with decreased fibrosis and lipogenesis [70]. Moreover, MIF ameliorates the symptoms of NASH and fibrosis via the CD74/AMPK pathway, which is shown by reduced accumulation of inflammation-related and oleic acid-elicited triglyceride. However, blockade of the MIF receptor inhibits the activity of AMPK and reverses these protective effects [54].

AFLD are also important factors that induce hepatic fibrosis. A corn oil–based diet protects against combined alcohol- and iron-induced mild steatohepatitis and portal–portal tract linkage fibrosis and increases the levels of AMPK, which is low in mice hepatic fibrosis models. This suggests that AMPK has a preventative role in fibrosis arising from alcoholic steatohepatitis [71]. Moderate obesity plus alcohol intake cause pericellular fibrosis and synergistic steatohepatitis in an alcohol dose-dependent manner whereas adiponectin increases the levels of AMPK and reverses these effects [72]. Jo and colleagues fed C57BL/6J mouse with a high-fat diet for 10 weeks and discovered that eugenol, an AMPK activator, ameliorated fatty acid-related hepatic fibrosis, concomitant with reduced expression of α-SMA, collagen I, and plasminogen activator inhibitor-1 (PAI-1). These results reveal that AMPK may represent a potential intervention of FLD-induced fibrosis [73].

### Biliary obstruction

Biliary obstruction is also a significant factor that induces fibrosis in both human and animal studies. Ursolic acid induces the expression of AMPK and attenuates the fibrotic response in the BDL mouse model, which corresponds with decreased collagen and α-SMA. However, silencing of AMPK reverses the ursolic acid-induced effects in cultured hepatocytes [74]. Rutin activates AMPK, ameliorates the deposition of ECM around newly formed bile ducts and bridging fibrosis in BDL rats, which is correlated with decreased levels of α-SMA and NF-κB [47]. These results suggest that AMPK can resist against biliary obstruction-induced hepatic fibrosis.

### CCl_4_

**Figure 2:**
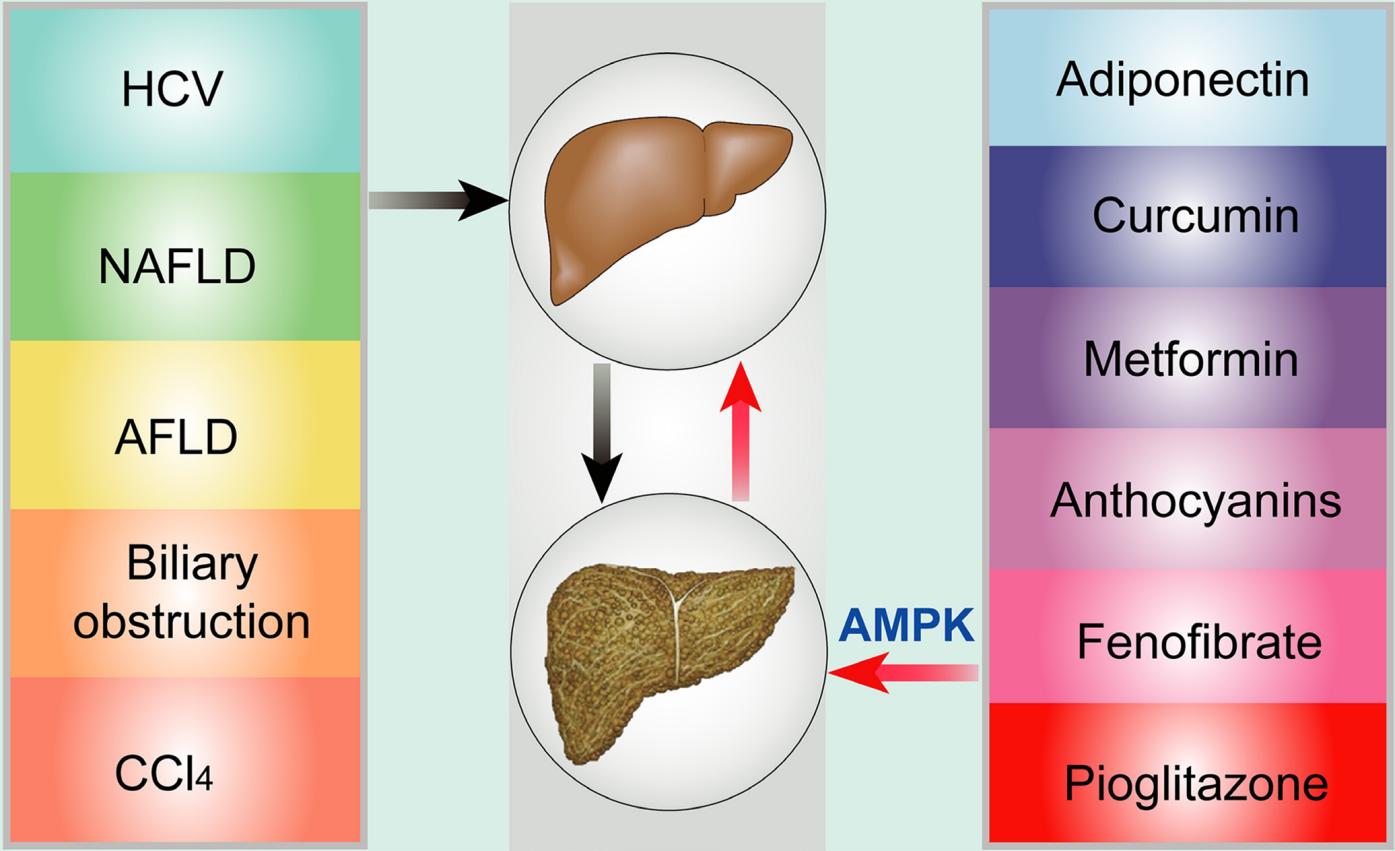
Risk factors and potential targets via AMPK throughout hepatic fibrosis Common risk factors of hepatic fibrosis include HCV, NAFLD, AFLD, biliary obstruction, and CCl4, which can induce hepatic fibrosis and inadequacy. Potential therapeutic targets via AMPK signaling consist of adiponectin, curcumin, metformin, anthocyanins, fenofibrate, and pioglitazone.

CCl_4_, also known as tetrachloromethane, is an organic compound that induces hepatic fibrosis in experimental animals. In the CCl_4_ mouse model, AMPK suppresses the expression of Nox4, TGF-β, and α-SMA and restrains the proliferation of HSC in a dose- and time-dependent manner [71]. ADP355 can attenuate CCl_4_-induced hepatic fibrosis via AMPK signaling. Histopathology has shown that chronic CCl_4_-treatment results in significant fibrosis and low levels of AMPK while ADP355 treatment reversed the fibrotic alteration, associated with increased AMPK and decreases in TGF-β1, connective tissue growth factor (CTGF), and the tissue inhibitor of metalloproteinase I (TIMP1) [45]. Moreover, melatonin reduces the production of collagen and α-SMA and attenuates CCl_4_-related hepatic fibrosis via AMPK signaling [52]. In short, these findings all demonstrate that AMPK can ameliorate fibrosis in different fibrosis models and act as a potent target for hepatic fibrosis. (Figure 2).

## THERAPEUTIC RESEARCH VIA TARGETING AMPK

### Adiponectin

Adiponectin, an adipocytokine encoded by the ADIPOQ gene, is involved in regulating glucose and fatty acid metabolism [75]. Adiponectin prevents the progression of fibrosis, which correlates with the restrained activity of HSC, reduced ECM, and increased activity of MMP-1 via the AMPK/JAK/STAT3 pathway whereas the adiponectin knockout mouse exhibit none of these effects [62]. Dong and colleagues reported that adiponectin attenuates HSC proliferation and migration but promotes apoptosis via an AMPK/iNOS/NO pathway, suggesting that AMPK is a potent anti-proliferative molecule against hepatic fibrosis [55]. Ramezani-Moghadam et al. discovered that adiponectin can inhibit liver fibrosis *in vivo* and limit HSC proliferation and migration *in vitro*. In addition, they analyzed serum from patients with liver fibrosis and discovered that levels of AMPK and adiponectin are low compared to healthy people, further confirming the protective actions of AMPK in fibrosis [39].

ADP355 attenuates the activation of HSC and the degree of fibrosis via AMPK signaling, as shown by reduced α-SMA, TGF-β, and TIMP1 in C57BL/6J mouse treated with CCl_4_ [45]. Moreover, ADP355 suppresses thioacetamide-induced activation of HSC and macrophages in the liver, thereby reducing the secretion of inflammatory cytokines and ameliorating hepatic fibrosis. These findings suggest that ADP355 is a potent agent acting against liver fibrosis via AMPK signaling.

### Curcumin

Curcumin is a well-known bioactive compound from turmeric [19]. Curcumin inhibits activation of HSC via AMPK/peroxisome proliferator activated receptor-γ coactivator-1α (PGC-1α) axis. Curcumin suppresses collagen I transcription but promotes superoxide dimutase-2 (SOD2) transcription. These data suggest that curcumin restrains HSC activation and liver fibrogenesis via AMPK signaling [61]. Curcumin eliminates effects of leptin on HSC activation and enhances AMPK activity, thereby increasing the levels of lipid droplet. This provides novel insights into mechanisms of curcumin in inhibiting leptin-induced HSC activation [52].

### Metformin

Metformin, marketed as Glucophage in the United States, is the first-line medication for diabetes [76]. Recent research has identified metformin as a potent anti-hepatic fibrosis compound [77]. Metformin induces the expression of bone morphogenetic protein and activin membrane–bound inhibitor (BAMBI) in quiescent HSC via AMPK activation, thereby ameliorating the degree of hepatic fibrosis [74]. Metformin significantly inhibits platelet-derived growth factor (PDGF)-stimulated proliferation, migration of human HSC, and secretion of monocyte chemoattractant protein-1 via AMPK signaling. However, knockdown of AMPK by gene silencing enhances the mitogenic effects of PDGF and fibrosis levels [56]. This result reveals that activation of AMPK by metformin suppresses the activation of HSC and attenuates hepatic fibrosis. In addition, metformin is able to promote the phosphorylation of AMPKα1 and inhibit the expression of type I α collagen and α-SMA, thereby preventing the HSC-mediated fibrogenic responses. However, this response is significantly attenuated by pretreatment with compound C [44]. Caligiuri and colleagues discovered that metformin mimics the effects of AICAR on HSC and effectively induces the phosphorylation of AMPK, which limits the secretion of type I procollagen. However, knockdown of AMPK by gene silencing increases the fibrosis degree, indicating that metformin ameliorates hepatic fibrosis at least partly via AMPK signaling [56].

### Other targets

Other studies have also demonstrated that AMPK is a potent target against hepatic fibrosis. Anthocyanins are able to improve the symptoms of NASH in mice receiving a methionine-choline-deficient (MCD) diet by activating the AMPK/PGC-1α signaling pathways. Meanwhile, anthocyanins suppress HSC activation and α-SMA and collagen I production in the NASH mouse, which is concomitant with a reduced degree of fibrosis [78]. Fenofibrate, an antilipemic agent which reduces both cholesterol and triglycerides in the blood, is able to activate AMPK and deliver anti-fibrotic actions [79]. In a C57BL/6 mouse steatohepatitis model induced by TGF-β or an MCD diet, fenofibrate promotes the expression of small heterodimer partner (SHP) and downregulates the levels of fibrosis [79]. Moreover, the thiazolidinedione, pioglitazone, improves hepatic fibrosis in rats with NASH by upregulating adiponectin expression and activating AMPK. This corresponds to the suppression of HSC and ECM overproduction, indicating that pioglitazone may be a drug candidate for hepatic fibrosis via AMPK signaling [59]. Resveratrol, an AMPK activator, is a natural polyphenol compound that has a broad spectrum of beneficial biological activities upon human health [80]. Kessoku’s group has reported that resveratrol ameliorates fibrosis and inflammation in a mouse model of NASH [81]. However, it remains unclear whether that effect is induced by AMPK activation. Notably, a recent study by Xu’s group reported that resveratrol can activate the AMPK signaling pathway and act as a potential therapeutic agent for NAFLD, but they did not provide further investigations on the internal mechanisms [82]. Thereby, whether resveratrol can treat CLD and hepatic fibrosis remains elusive. Together, the above-mentioned drugs all perform anti-fibrotic actions in the liver by activating AMPK. Further clinical studies are needed to confirm their safety and efficacy before clinical application.

## FURTHER PERSPECTIVE

As discussed above, AMPK is a powerful molecule that reduces hepatic fibrosis. Some studies have demonstrated that its anti-fibrotic actions are mediated by the inhibition of oxidative stress and inflammation. Oxidative stress seems to be an important process in the development of liver fibrosis [83–85]. Ursolic acid suppresses oxidative stress in hepatocytes through LKB1/AMPK/iNOS/Cox2 signaling in the mouse BDL model whereas ursolic acid fails to ameliorate fibrosis in the AMPK knockout mouse, as shown by enhanced fibrosis, lipid-oxidation, and iNOS/Cox-2 expression. This suggests that ursolic acid ameliorates oxidative stress-related hepatic fibrosis by activating LKB1/AMPK signaling [23]. Rutin treatment attenuates the reduction of catalase, Cu/Zn-SOD, and GSH in the rat BDL model via AMPK signaling, thereby ameliorating hepatic fibrosis [47]. In the section ‘Inflammatory Injury’, we have demonstrated that inflammation is a primary factor that induces hepatic fibrosis [23, 44, 46] and that some studies have revealed that hepatic fibrosis is simultaneously accompanied by oxidative stress and inflammation [47, 86]. This provides a new avenue for treating hepatic fibrosis via AMPK-related anti-oxidative and anti-inflammatory effects.

AMPK can suppress hepatic fibrosis via other mechanisms. Metabolic reprogramming is usually an important component of HSC. The most common reprogramming is aerobic glycolysis that allows HSC to acquire energy rapidly [87]. AMPK suppresses aerobic glycolysis and activation of HSC in a concentration-dependent manner, which is correlated with reduced lactate and ATP production, thereby blocking the normal energy supply of HSC and attenuating the fibrogenic process [88]. Growing evidence indicates a link between mitochondrial dysfunction and liver fibrogenesis [89, 90]. Melatonin enhances mitophagy and mitochondrial biogenesis and attenuates fibrosis in the rat CCl_4_ models by activating AMPK, as indicated by increased mitochondrial DNA and PTEN-induced putative kinase 1 (PINK1). This suggests that AMPK protects against liver fibrosis by promoting mitophagy and mitochondrial biogenesis [91]. Moreover, ursolic acid is able to suppress apoptosis of normal hepatocytes and delay the progression of fibrosis through LKB1/AMPK signaling, which reveals that protecting hepatocytes is also an effective method by which AMPK protects against fibrosis [23].

AMPK improves liver function in addition to its anti-fibrotic actions. In the mouse BDL model, ursolic acid-induced AMPK activation ameliorates liver function, which is evidenced by reduced alanine aminotransferase (ALT), aspartate aminotransferase (AST), alkaline phosphatase (ALP), triglyceride (TG), and total cholesterol (TC) whereas these effects are reversed in AMPK knockout mice [23]. The same effects were also shown in other studies [45, 50, 59, 72, 92], suggesting that AMPK is also a hepatoprotective drug that attenuates fibrosis and improves liver function.

Two faces of AMPK also exist for treating hepatic fibrosis. Several studies have shown that hypoxia and autophagy play key roles in the pathogenesis of hepatic fibrosis and the activation of HSC [93, 94]. Elevation of Ca^2+^ in the cytoplasm induced by hypoxic stress may activate the AMPK–mTOR and PKCθ pathway in HSC, leading to enhanced HSC autophagy and ultimately HSC activation. However, the autophagy inhibitor Bafilomycin A1 significantly attenuates the expression of α-SMA and fibrotic levels under hypoxic stress [95]. The study of Morais and colleagues reported that activated AMPK restrains hepatic fibrogenesis while fibrosis is not enhanced in AMPK knockout models [41], which is in conflict with other studies [39–41]. Silymarin, a listed hepatoprotective and anti-liver fibrosis drug, can improve symptoms of fibrosis and cirrhosis in rats by reducing levels of AMPK [96] while the authors did not give a proper explanation. We propose that the differences may result from the different models, *in vivo*/*vitro* experimentation, and AMPK concentrations. Additionally, some activators/agonists-induced AMPK activation is independent and some studies may exaggerate the positive roles of AMPK.

## CONCLUDING REMARKS

This review is devoted to the beneficial actions of AMPK in hepatic fibrosis. (Tables 1, 2) [97–102]. The processes of hepatic fibrosis include the following three phases: primary inflammatory injury, alterations of HSC, and ECM secretion; all of which can be delayed or reversed by AMPK. AMPK protects the liver against fibrosis under the conditions of HCV, FLD, biliary obstruction, and CCl_4_. In respect to these pro-fibrotic factors, we summarize anti-hepatic fibrotic drugs that act via AMPK signaling, such as adiponectin, curcumin, metformin, and others. The ability of AMPK to limits fibrosis may be partly attributed to the inhibition of oxidative stress, inflammation, aerobic glycolysis, as well as the promotion of mitophagy and mitochondrial biogenesis. In addition to its anti-fibrosis role, AMPK improves liver function and attenuates hepatocyte apoptosis, indicating that it acts as both a hepatoprotective and an anti-fibrotic molecule. Some objections against AMPK are also presented and are given a brief explanation. However, few studies have demonstrated the roles of AMPK in the clinic, which leads to an incomplete picture of AMPK. More clinical research is required to elucidate the potential of AMPK in hepatic fibrosis.

**Table 2:** Mechanisms of some AMPK activators

Activators	Mechanism	Specific	Directly	Reference
AMP	Direct binding to the γ subunit	Yes	Yes	[97]
AICAR	Acting as AMP analogs	No	Yes	[98]
LKB1	Promoting Thr172 phosphorylation of the α subunit	No	Yes	[99]
Adiponectin and ADP355	Promoting the phosphorylation of the α subunit	No	Yes	[45, 100]
Metformin and Resveratrol	Inhibiting ATP synthesis but promoting AMP synthesis	No	Yes	[101]
Rutin	Increasing the phosphorylated levels of AMPK	No	Yes	[47]
Thymoquinone	Increasing the phosphorylated levels of AMPK via LKB1 signaling	No	No	[51]
